# Biogenic carbon dots as dual mode luminescent sensors for hydrazine and ethanol

**DOI:** 10.1039/d5ra08528d

**Published:** 2026-04-24

**Authors:** Amina Zoha, Misbah Ejaz, Faisal Nawaz, Muhammad Ali Mohsin, Muhammad Nadeem Zafar, Zulfiqar Ali

**Affiliations:** a Department of Natural Sciences and Humanities, University of Engineering and Technology Lahore New Campus Lahore Pakistan faisal.nawaz@uet.edu.pk; b Department of Chemistry, Fatima Jinnah Women University Rawalpindi Pakistan; c Department of Chemistry, University of Gujrat Gujrat Pakistan

## Abstract

Carbon dots have gained considerable research attention owing to their excellent optical properties and environmental compatibility. In this study, a one-step hydrothermal synthesis of carbon dots (CDs) is reported using guava extract as a bio-based source. These as-prepared carbon dots were characterized by spectroscopic techniques including ultraviolet-visible spectroscopy, fluorescence spectroscopy, Fourier-transform infrared (FTIR) spectroscopy and X-ray diffraction (XRD). High-resolution transmission electron microscopy (HRTEM) analysis was used to study their structure and particle size. The characterization results revealed that the CDs had strong blue luminescence, a high density of oxygen-containing surface functional groups and an amorphous carbon structure. The UV-vis spectrum exhibited typical absorption peaks referred to as π–π* and n–π* transitions, and the fluorescence spectrum indicated the presence of excitation-dependent emission and optimal luminescence in the blue region. The presence of surface groups like hydroxyl, carboxyl and ether groups, as confirmed by FTIR analysis, imparts good hydrophilicity to the luminescent carbon dots. The synthesized carbon dots demonstrated excellent fluorescence-based sensing behavior, showing significant quenching in the presence of hydrazine and enhancement in luminescence upon ethanol exposure. The observed sensing behavior is attributed to the electron transfer interactions between the surface groups of the CDs and analytes.

## Abstract 2

This study explores synthesis and applications of carbon dots as dual-mode luminescent sensors for selective detection of hydrazine and ethanol.

## Graphical abstract

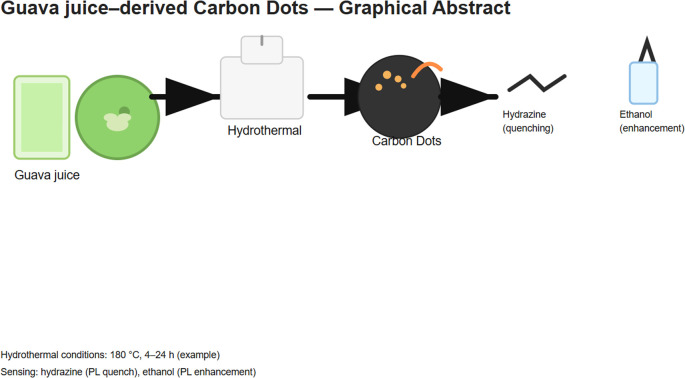

## 1 Introduction

Carbon dots (CDs), a special category of carbon-based luminescent nanomaterials typically sized under 10 nm, have become a prominent topic in materials science and nanotechnology since their accidental discovery in 2004 during the purification attempt of single-walled carbon nanotubes.^1^ Their low toxicity, high biocompatibility, excellent water dispersibility, and tunable optical properties make them more advantageous than traditional semiconductor quantum dots, thereby opening avenues for a wide range of applications. These applications include advanced bioimaging, highly sensitive chemical sensing, efficient photocatalysis, drug delivery systems and next-generation optoelectronic devices.^2–5^

CDs are generally synthesized either by top-down approaches (arc discharge, laser ablation, and electrochemical synthesis) or bottom-up approaches (chemical oxidation and thermal decomposition).^6,7^ However, harsh reaction conditions, expensive or toxic reagents, and the generation of unwanted byproducts in these methods motivate researchers to develop new cost-effective, sustainable and environmentally benign pathways.^8,9^

The green synthesis of CDs has therefore gained significant attention. Biomass-derived precursors, such as leaves, fruit extracts, peels and various agricultural wastes, are intrinsically rich in carbon, nitrogen, and oxygen heteroatoms.^10,11^ The presence of various organic compounds (*e.g.*, sugars, proteins, and organic acids) in these biomass sources provides the carbon backbone, and they function as self-doping and surface passivating agents, facilitating the formation of highly luminescent and functionalized CDs.^12–14^

Among various natural products, guava (*Psidium guajava*) is a particularly attractive precursor due to its rich contents of carbohydrate, protein and ascorbic acid. Ascorbic acid, a well-known antioxidant, acts as a carbonizing agent and facilitates the one-step synthesis of carbon dots without the need for any external acid or reducing agent. It promotes efficient carbonization and self-functionalization during hydrothermal processing, resulting in the formation of extremely stable and fluorescent carbon dots.^15–18^

Carbon dots can act as both reducing and oxidizing agents.^19^ Their ability to participate in electron transfer reactions, along with the presence of diverse surface functional groups, makes them excellent candidates for luminescent sensing applications.^20^

Herein, we report the synthesis of luminescent carbon dots *via* a one-pot hydrothermal route and their application as bimodal sensors for hydrazine and ethanol. The as-synthesized CDs exhibit contrasting responses toward these analytes, including luminescence quenching and enhancement, as well as red and blue shifts. The optical response of the CDs toward ethanol and hydrazine was found to be distinctly different. Upon incremental addition of ethanol (20–180 µL), the fluorescence intensity increased significantly, accompanied by a blue shift in the emission maximum, indicating surface passivation and reduced non-radiative recombination. In contrast, the addition of hydrazine (1–9 µL) induced pronounced quenching along with a red shift in emission, attributed to electron transfer and interactions with the emissive states of the CDs. This dual and opposite sensing behavior demonstrates the high sensitivity and tunable interaction potential of the prepared CDs toward chemically distinct analytes.^21–24^

## 2 Materials and methods

### 2.1 Materials

Ripe and fresh guava fruit (*Psidium guajava*) was locally gathered from a market in Lahore, Pakistan. All of the analytical-grade chemicals were used for cleaning and preparation and were obtained from Sigma-Aldrich. To maintain high purity and to avoid contamination, distilled water was used for all experimental procedures.

### 2.2 Synthesis of carbon dots

A hydrothermal carbonization process was designed for the synthesis of carbon dots from guava. A total of 100 gram of guava pulp was mixed with 200 mL of deionized water to make a homogenous slurry. A portion of the guava slurry was transferred into 100 mL Teflon-lined stainless-steel autoclaves using around 70% of the capacity of the autoclave. Multiple autoclaves were used under identical conditions. Then the autoclave was transferred to oven at 180 °C for 4 hours. The same procedure was repeated, only changing the hydrothermal treatment time to 12 and 24 hours, and two more samples were obtained. The autoclave was cooled to room temperature in air spontaneously after the hydrothermal reaction. The resulting dark brown solution was collected and filtered through Whatman filter paper (Grade 1) in order to eliminate any large uncarbonized particulates. The solution was then centrifuged for 20 minutes at 8000 rpm to ensure separation of any remaining larger carbonaceous agglomerates or precipitates. The clear supernatant solution, which contained the dispersed CDs, was then used for further characterization/applications.

## 3 Characterization techniques

A variety of sophisticated analytical methods were employed to determine the structural, chemical, and optical characteristics of the synthesized guava-derived carbon dots. The optical absorption properties of the carbon dots (CDs) were studied using a Shimadzu UV-1800 UV-vis spectrophotometer. Spectra were measured between *λ* = 200 and 800 nm using deionized water (DI) water as a reference blank to resolve the characteristic electronic transitions.^10^ The photoluminescent (PL) characteristics of the carbon dots, popularly denoted as CDs, have been studied using an Agilent Cary Eclipse fluorescence spectrophotometer. Emission spectra were accurately documented over a range of 350 and 600 nanometers, while the excitation wavelength was varied systematically from 300 to 420 nanometers in increments of 10 nanometers. This detailed research provided useful information regarding the luminescent characteristics of the CDs and revealed the nature of the phenomenon of excitation-dependent emission, while also pointing towards their possible aptness for a wide variety of photonic applications that may stand to benefit from their distinct traits.^25^

The functional groups found on the surface of the carbon dots, that are based on guava, were fully characterized using a Bruker Alpha Platinum ATR-FTIR spectrometer that is a very sophisticated piece of equipment for such measurements. Spectra were obtained over the wavenumber range between 4000 and 400 cm^−1^, and this measurement was performed using a resolution of 4 cm^−1^. This precise measurement helped to reveal useful information about the chemical bonds themselves as well as the several organic functionalities that are accessible for interaction at the surface. A very small aliquot of the CD solution was dried very carefully in order to prepare the sample for analysis.^3^ The structural characteristics and crystallinity aspects of the carbon dots (CDs) were studied using a Bruker D8 Advance X-ray diffractometer that operates using Cu Kα radiation (*λ* = 1.5418 Å). For the identification of whether the synthesized carbon dots are amorphous or crystalline in nature, the diffraction spectrum was captured between the values of 2*θ* of 10° to 80° using a step increment of 0.02° and a scanning rate of 2 seconds/step.^26^ HRTEM images of the as-synthesized carbon dots were obtained using JEM-F200 electron microscope to confirm the synthesis of carbon dots as well as to obtain the structural features of carbon dots.

## 4 Results and discussion

To investigate the effect of hydrothermal duration on the properties of CDs, the synthesis was carried out at different reaction times (4, 12, and 24 h). While no new functional groups were observed in the FTIR spectra, variations in UV-vis absorption and zeta potential indicate the gradual evolution of surface states and the degree of oxidation, highlighting the role of reaction time in tuning surface chemistry and colloidal stability.

### 4.1. UV-visible spectroscopy

**Fig. 1:**
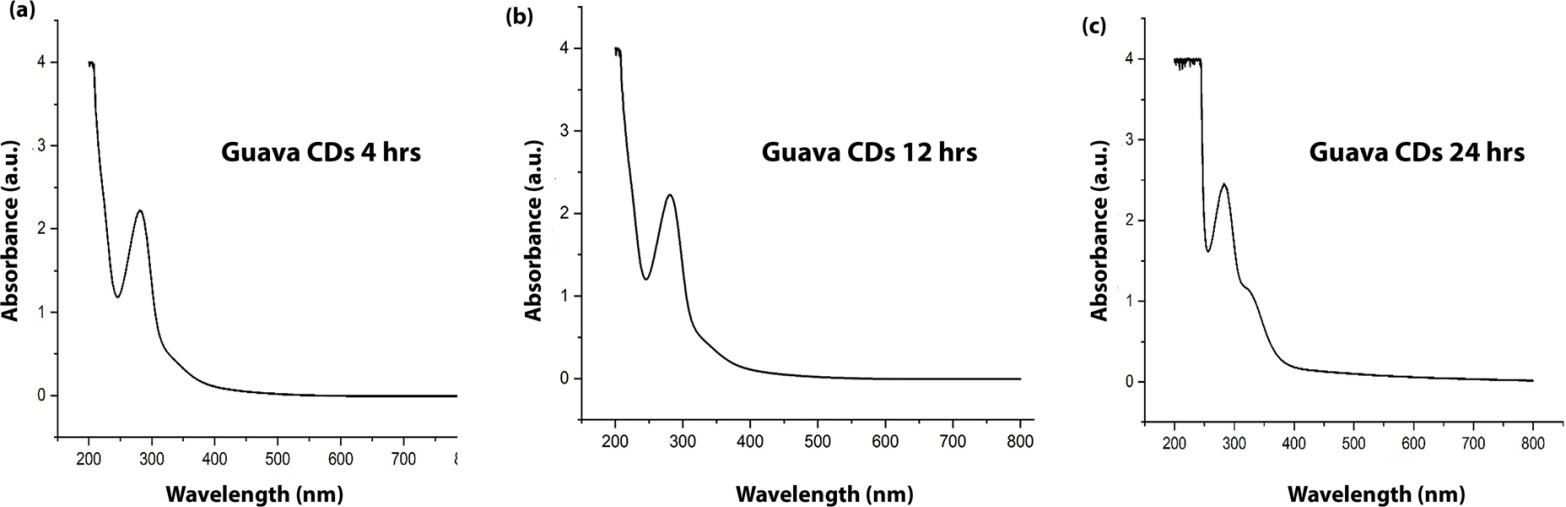
UV-vis spectra of the guava-derived carbon dots at 4 h (a), 12 h (b) and 24 h (c) under hydrothermal treatment times.

Three different samples of CDs were synthesized by varying the hydrothermal treatment time (4, 12 and 24 hours) at a fixed temperature of 180 °C. The UV-vis absorption spectrum of the as-synthesized CDs, as depicted in Fig. 1, exhibited two distinct absorption characteristics of carbon dots. A prominent and intense absorption peak was observed at approximately 275 nm. This peak is typically attributed to the π–π* transitions of the sp^2^ hybridized carbon atoms, which form the aromatic core of the carbon dots. This suggests the presence of conjugated carbon domains within the structure of the CDs. Additionally, a weaker shoulder or less defined peak was observed at around 320 nm. This feature is commonly ascribed to the n–π* transitions that arise from non-bonding electrons in a range of functional groups, particularly the carbonyl (C=O) and hydroxyl (–OH) groups that are extensively present on the surface of CDs made from biomass.^26^ The presence of these two absorption bands is in itself conclusive evidence that carbon dots with a rich electronic structure have indeed been successfully synthesized, as a result of their carbon core and surface passivation. The relatively broad distribution of these peaks is evidence of a variety of surface functionalization and carbon dot sizes. When we compare the absorption peaks obtained with varying hydrothermal treatment times, it is observed that with an increase in reaction time, a slight red shift is observed at the 275 nm peak, which may be attributed to extended conjugation within the carbon core or to enhanced graphitization.

As shown in Fig. 1, the absorption spectra of CDs synthesized at 4 h and 12 h are nearly identical, whereas the 24 h sample exhibits a noticeable enhancement in the n–π* transition band, indicating increased surface oxidation and the formation of additional surface defect states upon prolonged hydrothermal treatment.^27,28^

### 4.2 Photoluminescence studies and sensing applications

**Fig. 2:**
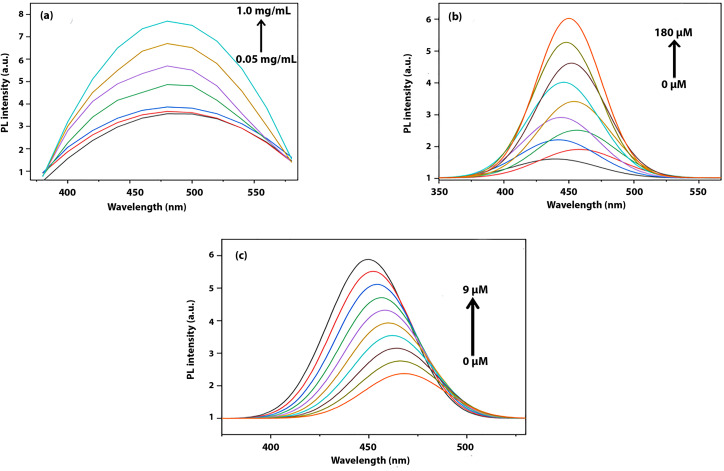
PL emission spectra of the guava CDs with (a) concentration variation, (b) ethanol addition (0–180 µL), and (c) hydrazine addition at *λ*_ex_ = 360 nm.

Fig. 2(a) presents the photoluminescence spectra of the carbon dots at varying concentrations (0.05–1 mg mL^−1^) under 360 nm excitation. The emission maximum remains centered around 470–480 nm, indicating stable emissive surface states, while changes in intensity are attributed to concentration-dependent interactions.^22^

Fig. 2(b) shows the sensing behavior of carbon dots towards ethanol. A quantitative increase in luminescence is observed upon incremental addition of ethanol (20–180 µM). A negligible peak shift indicates that the structure of the emissive sites remains intact and no new energy states are created. The enhancement in luminescence on addition of ethanol is likely the result of decreased surface trap density and improved radiative recombination efficiency due to hydrogen bond interactions between ethanol and polar surface groups.

As shown in Fig. 2(c), the addition of hydrazine (1–9 µM) shows contrasting trends. Hydrazine addition results in a progressive decrease in luminescence accompanied by a slight blue shift. This quenching behavior is attributed to electron or energy transfer from hydrazine to the CDs, which may lead to the deactivation of emissive sites. The associated blue shift upon addition of hydrazine is most likely linked to the interaction between hydrazine and surface molecules, leading to an overall increase in band gap of emissive sites. So, we may conclude from a luminescence behavior point of view that the opposite behavior of carbon dots with both analytes confirms their unique interaction with different analytes, showcasing their potential for unique bimodal sensors.^29–32^

As shown in Table 1, most reported carbon dot-based sensors are limited to single-analyte detection, typically exhibiting either fluorescence quenching or enhancement. In contrast, the present work demonstrates a unique bimodal sensing behavior toward hydrazine and ethanol, characterized by opposite fluorescence responses (quenching and enhancement), highlighting its potential for selective and versatile sensing applications.

**Table 1:** Comparison of the present work with already reported work

Material/source	Analyte	Detection method	Response type	Key features
Biomass-derived CDs (ref. 33)	Hydrazine	Fluorescence	Quenching	Green synthesis
Nitrogen doped CDs (ref. 34)	Hydrazine	Fluorescence	Quenching	High sensitivity
CDs (ref. 35)	Alcohols	Fluorescence	Enhancement	Fast response
CDs (ref. 36)	Ethanol	Optical	Shift	Surface interaction
This work	Ethanol and hydrazine	Fluorescence	Dual (quenching + enhancement)	Bimodal sensing and opposite response

### 4.3 FTIR analysis

**Fig. 3:**
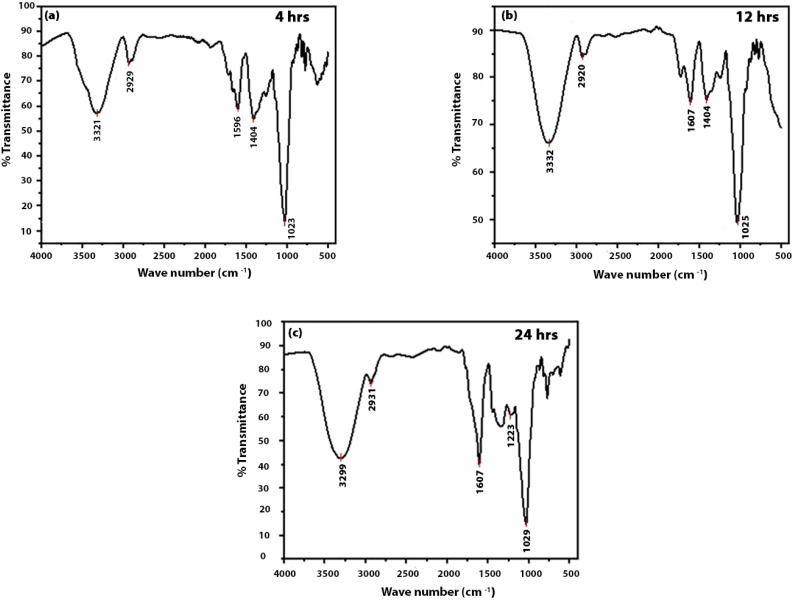
FTIR spectra of the guava-derived carbon dots with different hydrothermal treatment times such as 4 h (a), 12 h (b) and 24 h (c).

The Fourier-transform infrared (FTIR) spectrum (see Fig. 3) provides insight into the surface of the CDs extracted from guava. A strong and broad absorption band is observed around 3420 cm^−1^, which corresponds to the stretching vibration of O–H groups on the surface of the CDs and to adsorbed water molecules. This characteristic band confirms the presence of hydrophilic groups on the CDs' surface, which is consistent with the excellent water dispersibility of the as-synthesized carbon dots.^29^

A prominent peak observed near 1700 cm^−1^ is linked with the stretching vibration of the carbonyl group (C=O), indicating the presence of a number of carbonyl moieties like aldehydes, ketones, or carboxylic acids on the CD surface.^10^ The presence of carboxylic acid groups (–COOH) is particularly important because further surface functionalization can be facilitated by these groups. In addition, the presence of carboxylic acid groups can provide active sites for anchoring biomolecules in sensing or bioimaging applications. Other notable bands include stretching vibrations at 1610 cm^−1^ which may be attributed to the C=C stretching vibrations of the sp^2^ hybridized carbon core or to bending vibrations of adsorbed water. Peaks near 1050 cm^−1^ further indicate the stretching vibrations of the C–O bonds, which indicate the functionalities of the C–O–C (ether) and the C–OH (alcohol).^30^ The CDs' photoluminescent and colloidal stability in aqueous media may be attributed to the presence of such oxygen-containing functional groups on the surface. The coexistence of carbonyl, hydroxyl and ether functional groups confirms the presence of surface-passivating groups and brings out the potential of carbon dots for diverse applications.

With the change in reaction time, only minor changes in relative band intensities are observed, which suggest that no new functional groups appear, though surface sites may be suggested to evolve.

### 4.4 XRD analysis

**Fig. 4:**
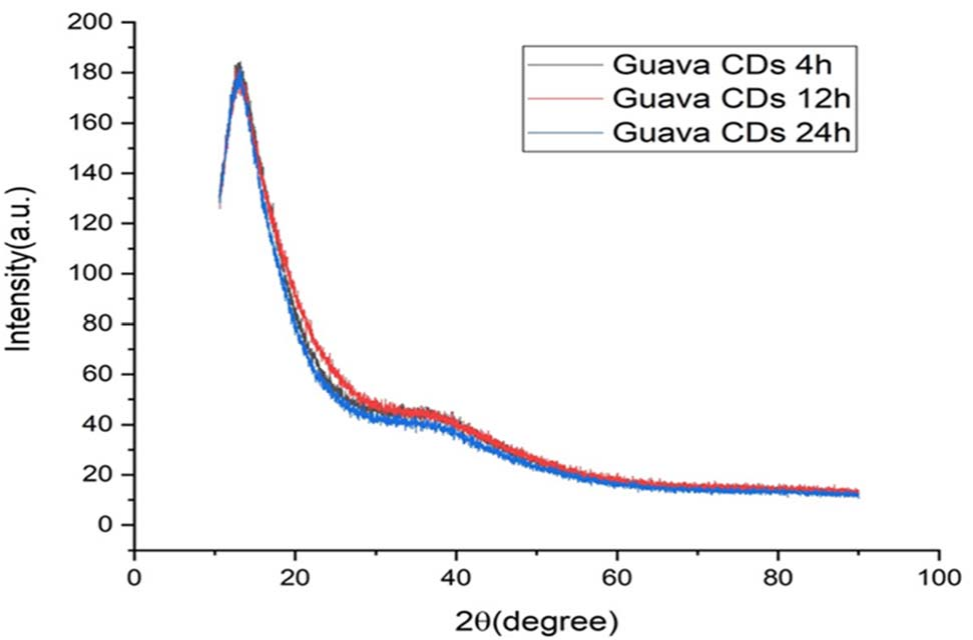
XRD pattern of the guava-derived carbon dots.

Fig. 4 presents the X-ray diffraction spectrum. It offers key insights into the internal structure and amorphous properties of the carbon dots derived from guava. A prominent diffraction peak has been found between 2*θ* = 24°–26°, which is a typical signature for amorphous carbon structures.^37^ The presence of this broad peak indicates that the CDs possess a graphite-like or graphene-like composition but lack long-range order, possibly due to stacking of graphitic layers and thereby a predominantly disordered state in the atomic level.

The broad diffraction peak corresponds to the (002) graphitic carbon plane; it is, however, severely broadened due to the small size and partially disordered stacking of the graphitic layers. From Bragg's law (2*d* × sin *θ* = *nλ*), the calculated spacing between the layers (*d*-spacing) is approximately 0.35 nm, slightly larger than that of bulk graphite (0.334 nm). This enhanced spacing for carbon dots is due to the presence of a number of defects, surface functional groups, and structural irregularities within the carbon lattice. Such partially ordered structures are commonly observed for carbon dots synthesized through the hydrothermal procedure using biomass precursors, where the carbonization procedure results in partial graphitization but without complete crystallization. The interaction between this local crystallinity and high surface functionalization plays a critical role in defining the particular optical properties of the carbon dots.

A comparative analysis of structural changes in CDs under different reaction times was also performed. As evident in Fig. 4, a broad amorphous hump at around 20° (2*θ*) shows that all CDs have the same basic structure.

### 4.5 TEM analysis

**Fig. 5:**
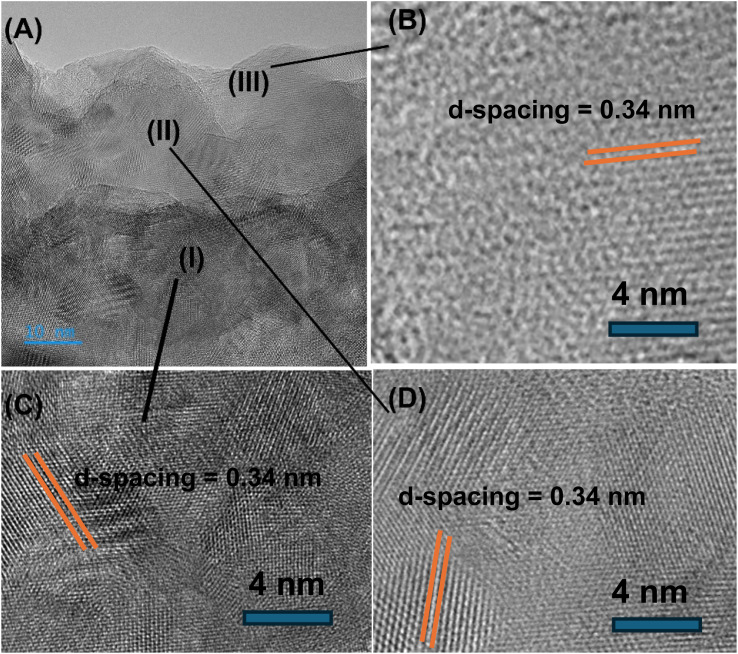
High resolution transmision electron microscopy (HRTEM) monograph of guava carbon dots (A), and three distinctive regions marked as (I), (II) and ((III) are shown in (B), (C) and (D).

Fig. 5 reveals that the crystallinity is maintained throughout the carbon-dot structure, and three distinctive regions are observed (*i.e.*, marked as (I) (II) and (III)) having the same *d*-spacing value of 0.34 nm. A symmetrical crystalline structure of carbon-dots provides enhanced photo–luminescent activity.

### 4.6 Zeta potential measurements

The zeta potential measurements of the synthesized carbon dots revealed negative surface charges for all samples, with values ranging from −21 to −35 mV (Table 2). This negative surface charge originates from the presence of oxygen-containing functional groups such as hydroxyl and carboxyl moieties. Notably, samples synthesized at longer reaction durations exhibited more negative zeta potential values (−35 mV), indicating enhanced colloidal stability due to stronger electrostatic repulsion, whereas samples obtained at shorter durations (−21 and −24 mV) showed comparatively moderate stability in aqueous media.

**Table 2:** Zeta potentials of the carbon dots at various reaction durations

Sr. no.	Reaction time	Zeta potential (mV)
1	4 hours	−21
2	12 hours	−24
3	24 hours	−35

The observed variation in zeta potential values suggests a progressive increase in surface oxidation and functionalization with reaction time, which is also observed from UV-visible spectra. This increase in negative surface charge reflects the formation of a greater number of surface defect sites and oxygenated groups, which play a crucial role in improving dispersion stability as well as facilitating interactions with analyte molecules during sensing applications.

## 5 Conclusion

The current work successfully demonstrates a green and sustainable route for the synthesis of highly fluorescent carbon dots using a simple hydrothermal process that involves the utilization of guava fruit extract as a green precursor. Comprehensive characterization done through UV-vis, fluorescence, FESEM, HRTEM, FTIR, and XRD studies confirmed the synthesis of stable carbon dots having promising structural and emission attributes, highlighting their prospects for utilization in a variety of high-end applications. The existence of conjugated carbon cores and large quantities of surface oxygen functionalities is confirmed by UV-visible spectroscopy. The surface of the CDs is found to be enriched with hydroxyl, carbonyl and ether groups, which make them water soluble and highly reactive in various chemical reactions, as confirmed by FTIR analysis. The XRD pattern was found to have an amorphous structure, which is characteristic of biomass-derived carbon nanomaterials. These as-synthesized CDs showed a distinct behavior towards ethanol and hydrazine as bimodal sensors.

Future works will be focused on optimizing the synthesis parameters for improving quantum yield, achieving fine size control and determining their practical application in photonic and sensing devices.

## Conflicts of interest

There are no conflicts to declare.

## Data Availability

Data are available on request.
